# Anti-Zic4 Paraneoplastic Cerebellar Degeneration With Mesial Temporal Lobe Hyperintensity in a Patient With Papillary Thyroid Carcinoma: A Case Report and Review of the Literature

**DOI:** 10.7759/cureus.36164

**Published:** 2023-03-15

**Authors:** Saad Abuzahra, Ahmad Abuhassan, Mosab Maree, Maha Makkawi

**Affiliations:** 1 Faculty of Medicine and Health Sciences, An-Najah National University, Nablus, PSE; 2 Department of Neurology, An-Najah National University Hospital, Nablus, PSE; 3 Department of Radiology, An-Najah National University Hospital, Nablus, PSE; 4 Department of Pathology, An-Najah National University Hospital, Nablus, PSE

**Keywords:** case report, onconeural antibodies, paraneoplastic neurological syndrome, paraneoplastic cerebellar degeneration, papillary thyroid carcinoma

## Abstract

Paraneoplastic cerebellar degeneration (PCD), a subtype of paraneoplastic neurological syndromes (PNSs), is a rare autoimmune neurologic syndrome that usually develops secondary to an underlying malignancy. We present a 49-year-old patient that developed PCD secondary to occult papillary thyroid carcinoma. The patient had progressive difficulty ambulating for 3 years. A neurological exam revealed signs of cerebellar syndrome. Brain magnetic resonance imaging (MRI) showed significant cerebellar atrophy and mesial temporal lobe hyperintensity. Immunological testing was highly positive for anti-CV2 and anti-Zic4 onconeural antibodies. Positron emission tomography (PET)/Computerized tomography (CT) scan revealed significant hypermetabolic uptake of F-18 fluorodeoxyglucose (FGD) by a left thyroid nodule. Histological examination of the nodule was positive for papillary thyroid carcinoma, confirming the diagnosis of PCD. A trial of high-dose methylprednisolone failed to improve the patient’s symptoms. This case highlights the importance of maintaining high suspicion for PCD while investigating cases of cerebellar degeneration. Early detection is essential to prevent irreversible damage in affected patients.

## Introduction

Paraneoplastic cerebellar degeneration (PCD), a subtype of paraneoplastic neurological syndromes (PNSs), are uncommon syndromes that occur secondary to different tumors. PNSs are caused by onconeural antibodies that cross-react with self-antigens in the nervous system [1]. PCD is one syndrome of PNSs. Humoral autoimmune destruction of the cerebellar Purkinje cells seems to cause the development of PCD. Although PCD has been associated with various malignancies, it is most commonly associated with breast and gynecological tumors [2, 3]. In this paper, we report a case of anti-Zic4 PCD with mesial temporal lobe hyperintensity in a patient with papillary thyroid carcinoma.

## Case presentation

A 49-year-old woman presented to the hospital complaining of progressive gait instability over three years. This limited the patient’s ability to ambulate and perform daily activities. The patient had no significant medical, surgical, or family history of similar illness.

On neurological exam, the patient was alert and oriented to person, place, and time with a GCS of 15. Her speech was fluent with intact naming, repetition, and comprehension. Cranial nerves II-XII were intact. Her motor exam was normal, with muscle strength of 5/5. The patient had brisk reflexes (+3) throughout. Positive Babinski sign was present bilaterally. Her sensation was intact and there were no deficits. Finger-to-nose and heel-to-shin test were abnormal, indicative of bilateral dysmetria. The patient had a cerebellar ataxic gait. 

**Figure 1:**
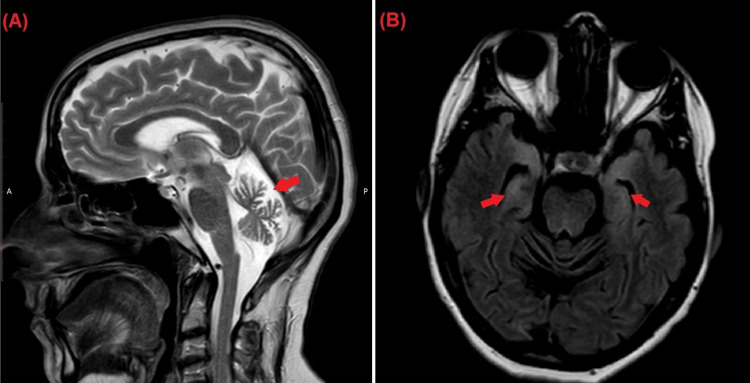
Sagittal T2-weighted images MRI (A) shows significant cerebellar atrophy manifested by volume loss and widening of the CSF space (arrow); Axial FLAIR MRI (B) shows bilateral asymmetrical increased signal intensity of both mesial temporal lobes (arrows). MRI, magnetic resonance imaging; CSF, cerebrospinal fluid

Routine blood tests, including inflammatory markers, thyroid function tests, vitamin E levels, and vitamin B12 levels were normal. Lumber puncture and analysis of the cerebrospinal fluid (CSF) were unremarkable. MRI imaging found significant cerebellum degeneration (Figure 1A) and bilateral asymmetrical hyperintensity in the mesial temporal lobes (Figure 1B). Immunoassay for onconeural antibodies found high titers of anti-CV2 and anti-Zic4 antibodies. 

**Figure 2:**
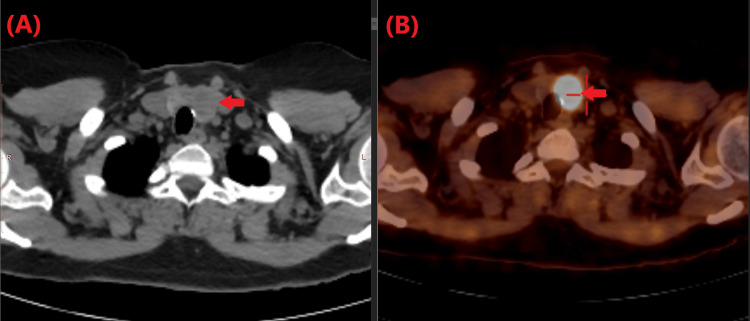
CT scan (A)/ PET scan (B) at the level of thyroid gland showing 1.8 cm left thyroid nodule (arrows) with significantly increased F-18 FDG uptake. CT,  computerized tomography; PET, positron emission tomography; FDG, fluorodeoxyglucose

**Figure 3:**
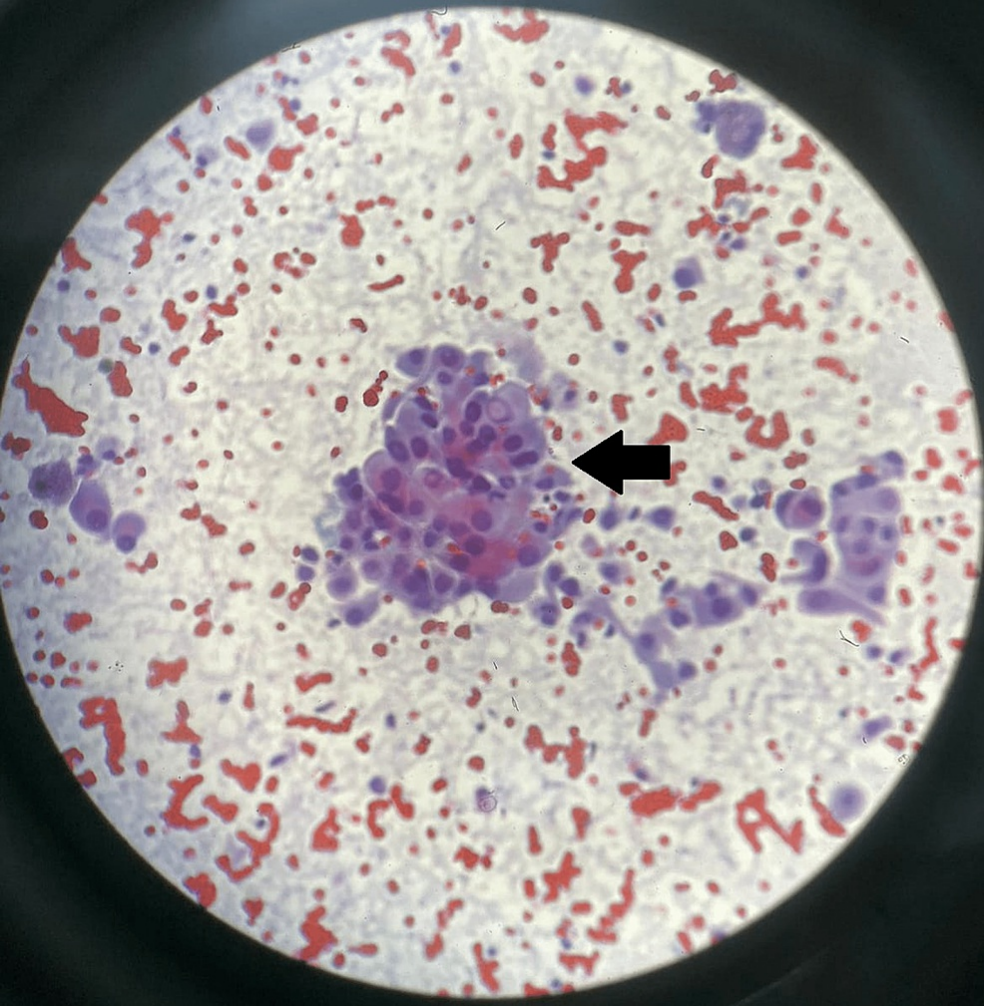
Histological examination shows sheets of follicular epithelial cells with large nuclei, nuclear overlapping, and many intranuclear inclusions (arrow). These findings are indicative of papillary thyroid carcinoma.

CT/PET of the neck, chest, and abdomen demonstrated intense hypermetabolic uptake of F-18 FDG in a left thyroid nodule (Figures 2A, 2B). The patient underwent ultrasound-guided fine needle aspiration (FNA) of the nodule. Histopathology report revealed malignant cells, suggestive of papillary thyroid carcinoma. The FNA specimen had a score of six (VI) on the Bethesda system (Figure 3).

Despite a trial of high-dose methylprednisolone at a dose of 1g/day for 5 days, the patient's symptoms did not improve. The patient was then referred to a specialized oncology center for further treatment, but the patient lost follow-up.

## Discussion

PNSs are presenting a new challenge for physicians as recognition of PNSs has been increasing in the literature. However, the incidence of PNSs remains low, affecting less than 1% of cancer patients [4]. Researchers have not yet understood the exact pathophysiology of PNSs. However, the cross-reactive onconeural antibodies seem to present the most plausible explanation [5]. PNSs become clinically overt prior to the cancer diagnosis [6]. Thus, early recognition and investigation may enhance the rate of cancer detection and survival in affected patients.

PCD, a subtype of PNSs, is a rare manifestation of malignant tumors. Despite that, it still resembles the second most common cause of paraneoplastic syndromes and immune-mediated cerebellar ataxia. PCD seems to occur more frequently with certain malignancies, including gynecologic tumors, breast cancer, lung cancer, and Hodgkin’s lymphoma [7]. In contrast, researchers have rarely reported PCD occurring with papillary thyroid carcinoma.

In 2014, Gratwicke et al. reported a case of a 71-year-old patient with signs of cerebellar dysfunction. The patient received a diagnosis of PCD and papillary thyroid carcinoma [8]. Additionally, Kroiss et al. described a case of PCD in a 57-year-old patient with papillary thyroid cancer. The patient complained of worsening symptoms associated with cerebellar dysfunction. Papillary thyroid carcinoma was then detected on the PET scan [9]. Ayas et al. reported a case of a 47-year-old patient that was diagnosed with PCD and papillary thyroid carcinoma [10]. 

PCD can present with either an acute or sub-acute onset [11]. In the cases reported above, only Gratwicke et al. reported rapid progression of symptoms over 4 months. However, similar to our case, Ayas et al. reported symptoms that have been slowly progressing over two years prior to diagnosis [8,10]. The pattern of slow insidious onset reported in these two cases is atypical for PCD. This may suggest that papillary thyroid carcinoma is associated with a slower onset of PCD.

Similar to the reported cases, our patient had no symptoms or signs indicative of thyroid carcinoma. This may hinder the physician's ability to diagnose the patient correctly early on. Therefore, it is important to test for PCD in cases with unexplained cerebellar degeneration. Thyroid cancer was first detected on a PET scan in all reported cases. Thus, a PET scan can serve as an important screening tool for cancer in cases with suspected PCD [8-10]. Specific diagnostic tests should be conducted to confirm the diagnosis if a mass is detected. 

Autoantibody detection is important in assessing PCD to help establish a diagnosis, predict cancer associated with that antibody, and allocate the specific treatment needed [12]. Therefore, physicians may use autoantibody testing in patients with unexplained neurological symptoms. Our patient had a normal workup with no obvious cause for the neurological symptoms. Therefore, an extensive immunoassay for onconeural antibodies was done. Immunoassay displayed high titers of anti-CV2 and anti-Zic4. In contrast to anti-Zic4, anti-CV2 autoantibody has been strongly associated with PCD. To our knowledge, this is the first case to report anti-Zic4 antibodies in a patient with PCD and papillary thyroid carcinoma [12].

Brain imaging can help diagnose PCD. Cerebellar atrophy is the most common abnormal finding seen on MRI imaging in PCD [12]. Besides cerebellar atrophy, our patient demonstrated hyperintensity in both mesial temporal lobes. Hyperintensity of the mesial temporal lobe is seen in cases of limbic encephalitis. However, the patient did not exhibit any symptoms indicative of limbic encephalitis such as working memory deficits, seizures, or psychiatric symptoms [13]. Thus, a diagnosis of isolated PCD is more likely. However, abnormal findings on imaging are not a must. Up to 75% of cases have no significant MRI findings, suggesting that abnormalities may not be visible until late in the disease's course [14]. This suggests a possible temporal relationship between the course of the disease and the severity of findings on imaging.

Overall, PNSs and PCD have unfavorable outcomes and prognoses. Early detection is an important predictor of survival and reversibility of symptoms. Treatment revolves around treating the underlying malignancy if found. Immunosuppression can be used to alleviate symptoms. Thus, methylprednisolone 1g per day for 5 days is the first-line immunosuppressive therapy for affected patients. Additionally, physicians should consider intravenous immunoglobulins or plasma exchange on a case-by-case basis [12].

## Conclusions

PCD can cause significant disability in affected patients. We report a rare case of anti-Zic4 PCD associated with papillary thyroid cancer. Treatment of the malignant tumor remains the mainstay of treatment, with immunosuppressive therapy being used for symptomatic relief. However, early detection and treatment are vital in preventing the significant progression of PCD. It is important to emphasize that physicians must maintain a high level of awareness for PNSs while investigating cases with unexplained neurological symptoms.
